# Prenatal genotype-phenotype correlations in four fetuses with Rubinstein-Taybi syndrome due to EP300 mutations: a case series and literature review

**DOI:** 10.3389/fgene.2026.1867006

**Published:** 2026-07-15

**Authors:** Tingting Ge, Jun Qi, Xiyuan Deng, Xiaoyu Song, Ling Hui, Yongwen Cao, Yupei Wang, Chuan Zhang, Xiaojuan Lin

**Affiliations:** 1 Key Laboratory of Maternal-Fetal Medicine and Reproductive Protection of Gansu Province, Prenatal Diagnosis Center, Gansu Provincial Maternity and Child-care Hospital (Gansu Provincial Central Hospital), Lanzhou, China; 2 Center for Medical Genetics, Gansu Provincial Maternity and Child-care Hospital (Gansu Provincial Central Hospital), Gansu Provincial Clinical Research Center for Birth Defects and Rare Diseases, Lanzhou, China; 3 Planning and Finance Department, Gansu Provincial Maternity and Child-care Hospital (Gansu Provincial Central Hospital), Lanzhou, China

**Keywords:** EP300, fetuse, genotype-phenotype, prenatal, rubinstein-taybi syndrome

## Abstract

**Objective:**

This study aims to delineate the prenatal ultrasound characteristics of four cases of Rubinstein-Taybi syndrome type 2 (RSTS2) and explore potential associations with this condition as detected through prenatal ultrasound.

**Methods:**

Whole exome sequencing (WES) and Sanger sequencing were conducted on four fetuses diagnosed with RSTS2. Prenatal ultrasound data were systematically collected and analyzed.

**Results:**

All four fetuses were found to have previously unreported *EP300* variants, which were absent from ClinVar and the Human Gene Mutation Database (HGMD). The prenatal ultrasound findings were diverse; in the second trimester, two fetuses exhibited growth parameters below minus two standard deviations (M-2SD). One fetus showed an abnormal foot posture and thickened plantar skin, another was found to have exencephaly in the first trimester, and one case presented with fetal rhabdomyoma.

**Conclusion:**

This study prenatal ultrasound phenotypes associated with *EP300* variants may add to the phenotypic profile of RSTS2 and could serve as a reference for prenatal diagnosis and counseling.

## 1 Introduction

Rubinstein-Taybi Syndrome (RSTS), initially identified in 1963, is a rare congenital disorder with an incidence ranging from 1 in 125,000 to 1 in 100,000 individuals. Also referred to as Broad Thumb-Hallux Syndrome, RSTS is characterized by a wide spectrum of clinical manifestations (Rubinstein and Taybi, 1963). It is an autosomal dominant genetic disorder, although the majority of cases arise as *de novo* variants.

RSTS is classified into two subtypes, type 1 (RSTS1, OMIM #180849) and type 2 (RSTS2, OMIM #613684). RSTS1 is attributed to variants in the *CREBBP*, accounting for approximately 50%–70% of cases, whereas RSTS2 is associated with mutations in the *EP300*, representing only 5%–8% of cases (Lacombe et al., 2024; López et al., 2018; Roelfsema et al., 2005). While both subtypes exhibit classical phenotypic features, distinct differences exist between them. Patients with RSTS2 generally present with milder facial features, less pronounced radial deviation of the thumb, infrequent keloid formation, and higher average cognitive levels (Hamilton et al., 2016). However, RSTS2 is linked to increased incidences of maternal preeclampsia, fetal growth restriction, and microcephaly compared to RSTS1 (Cohen et al., 2020; Fergelot et al., 2016; Negri et al., 2016).

The primary pathogenic mechanism underlying RSTS involves loss-of-function heterozygous mutations in either the *CREBBP*, which encodes the transcriptional coactivator CBP, or the *EP300*, which encodes the homologous protein p300 (López et al., 2018). The *CREBBP* is located on 16p13.3, spans approximately 155Kb, and contains 31 exons. Similarly, the *EP300* is located on 22q13.2, extends over approximately 152 kilobases, and also contains 31 exons. CBP and p300 exhibit a high degree of structural homology, particularly within their functional domains, with approximately 63% amino acid sequence homology and 73% similarity. As critical histone acetyltransferases and transcriptional coactivators, CBP and p300 are integral to the regulation of gene expression, cell differentiation, and embryonic development. The core pathogenesis of RSTS is attributed to haploinsufficiency, wherein the inactivation of a single allele results in a reduced dosage of CBP/p300 protein. This reduction significantly disrupts the chromatin remodeling and transcriptional regulatory networks mediated by CBP/p300, ultimately leading to the characteristic craniofacial deformities, broad thumbs and toes, variable structural abnormalities, and intellectual disability associated with RSTS (Cohen et al., 2020; Fergelot et al., 2016; Goodman and Smolik, 2000; Matasariu et al., 2025).

Currently, most phenotypic descriptions of RSTS pertain to postnatal manifestations, with prenatal reports predominantly associated with *CREBBP* variants. At present, there are few cases of prenatal diagnosis of RSTS2 caused by *EP300* variants. Therefore, we analyzed the genetic characteristics of 4 fetuses with RSTS2 and their prenatal ultrasound features, with the aim of providing theoretical basis for the ultrasound diagnosis of this disease in the future.

## 2 Methods

We recruited four RSTS2 cases that underwent invasive prenatal procedures at Gansu Provincial Maternity and Child-care Hospital (Gansu Provincial Central Hospital) between 2021 and 2025. The pregnant women aged 27–34 years, with a mean age of 31.25 ± 2.99 years (Table 1). Cases 1–3 underwent prenatal genetic testing via amniocentesis. Case 4, molecular confirmation was obtained postnatally from neonatal venous blood after emergency cesarean delivery. No prenatal genetic testing was performed due to the emergent nature of delivery and the absence of a clear prenatal suspicion for RSTS2. As the ultrasound examination results of 4 fetuses were abnormal (Table 1), after genetic counseling, all of them were required to undergo WES testing to clarify the genetic cause. This study was undertaken according to the tenets of the Declaration of Bange and Niedhart, 2008 and its later amendments. The study protocol was approved by the Ethics Committee of the Gansu Provincial Maternity and Child-care Hospital (Gansu Provincial Central Hospital) (2021) GSFY Lunshen No. 65. Written informed consent was obtained from all study participants.

**TABLE 1:** Clinical data and prenatal ultrasound examination results of 4 case.

Case	MA (yrs)	Gestation at diagnosis (W)	Ultrasound findings	Pregnancy outcome
1	34	12	Exencephaly	TOP
2	27	24	Rhabdomyoma	TOP
3	32	27	FL: M - 2.1SD HL: M - 3.1SD Bilateral thick plantar surfaces Bilateral talipes equinovarus	TOP
4	32	Newborn	The smaller fetus of DCDA 24 weeks HC, FL, HL < M - 2SD 35+5 weeks Emergency cesarean section was performed due to absent end-diastolic flow in the umbilical artery	Died at 2 months after birth
FL: Femur Length; HL: Humerus length; TOP: Termination of pregnancy; DCDA: Dichorionic diamniotic twin; HC: Head circumference				

Ultrasound examinations were performed using the Voluson E10 color Doppler ultrasound diagnostic instrument, equipped with a C1-6 probe operating within a frequency range of 4–8 MHz. The pregnant participants were positioned in the supine position, and the ultrasound examinations were conducted by sonographers with over 5 years of experience in prenatal ultrasound screening. In accordance with the Chinese Expert Consensus on Fetal Growth Restriction (2019 edition)[9] (Fetal Medicine Subgroup, Society of Perinatal Medicine, Chinese Medical Association, Obstetrics Subgroup, Society of Obstetrics and Gynecology, Chinese Medical Association, 2019), the NICHD Asian population curve was recommended for assessing fetal size in the Chinese population. Amniocentesis was performed under sterile conditions to collect 30 mL of amniotic fluid. Concurrently, peripheral blood samples (2–3 mL) were obtained from both parents for genomic DNA extraction. Subsequent analyses included WES, followed by Sanger sequencing for variant validation.

WES was performed on the BGISEQ-2000 platform (BGI, Shenzhen, China) using the BGI Exome Capture V1 kit (BGI, Shenzhen, China). The average sequencing depth was 200x, with 98.5% of target regions achieving ≥30x coverage and 99% achieving ≥20x coverage. Variant calling for single-nucleotide polymorphisms (SNPs) and insertions/deletions (Indels) was conducted following the GATK best practices workflow. Short-read sequencing data were aligned to the reference genome GRCh37/hg19 using BWA-MEM, followed by duplicate marking, base quality score recalibration, and variant discovery using Haplotype Caller. Final variants were filtered based on recommended quality metrics. Copy number variant (CNV) analysis from exome data was applied in this study,the resolution was>100 kb. Detected variants were annotated and interpreted according to the ACMG guidelines (Richards et al., 2015).

## 3 Results

In case 1, prenatal ultrasound examination at 12 weeks revealed fetal exencephaly. In case 2, prenatal ultrasound examination at 24 weeks revealed fetal cardiac rhabdomyoma. In case 3, prenatal ultrasound examination at 27 weeks revealed fetal femur length (FL) below M-2SD, humerus length (HL) at M-3.1SD, mildly thickened bilateral plantar surfaces, and bilateral talipes equinovarus. In case 4, fetus of dichorionic diamniotic twin (DCDA) through assisted reproduction, with no significant abnormalities detected at first trimester, however, ultrasound at 24 weeks revealed that the head circumference (HC), FL, and HL below M-2SD for gestational age. At 35+5 weeks of gestation, an emergency cesarean section was performed due to absent end-diastolic flow in the umbilical artery. WES was not performed due to lack of prenatal indications. After birth, whole exome sequencing via venous blood identified a pathogenic variant in the EP300 gene. Regarding pregnancy and follow-up outcomes. Cases 1–3 opted for pregnancy termination following the receipt of genetic results, and case 4 died at 2 months after birth (Table 1). After WES, Sanger Sequencing and Kinship analysis, all four affected fetuses (proband) were identified *de novo* pathogenic/likely pathogenic/Uncertain Significance in *EP300* (NM_001429.3) (Table 2). All the four *EP300* variants were novel and have not been reported in the ClinVar(www.ncbi. nlm. nih.gov/clinvar/) and HGMD (www.hgmd.cf.ac.uk/) databases (Table 2). Three cases received prenatal confirmation, while one case was confirmed during the neonatal period.

**TABLE 2:** Genetic diagnosis data of 4 case.

Case	Sample type	Detection method	Position	Nucleotide change	Amino acid change	Variant type	Pathogenic assessments	Evidence of pathogenicity
1	AF	Trio-WES	chr22:41572266	c.4795delC	p.Arg1599fs*25	Frameshift	Pathogenic	PVS1+PS2+PM2_Supporting
2	AF	Trio-WES	chr22:41574531	c.6816delT	p.Val2273Phefs*6	Frameshift	Likely pathogenic	PVS1_Moderate + PS2+PM2_Supporting
3	AF	Trio-WES	chr22:41523695	c.1111A>G	p.Thr371Ala	Missense	Likely pathogenic	PS2+PP3#+PM2_Supporting_Supporting
4	V	Trio-WES	chr22:41545967	c.2582C>T	p.Ala861Val	Missense	Uncertain significance	PS2+PM2_Supporting
AF: Amniotic fluid; V: Venous blood; Trio-WES: Trio whole exome sequencing								

## 4 Discussion

Previous research has indicated that prenatal growth restriction and microcephaly are prevalent among patients with RSTS2. For instance, Fergelot et al. reported an incidence of 42% for prenatal growth restriction and 87% (45/52) for microcephaly (Fergelot et al., 2016). Similarly, Cohen et al. documented a prevalence of 50% for prenatal growth restriction and 83% for microcephaly (Cohen et al., 2020). Furthermore, Negri G et al. found that two out of six RSTS2 patients exhibited prenatal growth restriction, while four experienced postnatal growth retardation (Negri et al., 2015). These findings underscore the critical importance of prenatal identification. Consequently, the present study examined four fetuses with prenatally diagnosed *EP300* abnormalities. Among these, 50% (2/4) demonstrated growth restriction, aligning with previous reports. In both cases, the HL and FL were below the normal range for the respective gestational age, and HC in one case was also below the normal range for the gestational age. Additionally, one case exhibited bilateral abnormal posture and plantar skin thickening, another was diagnosed with severe exencephaly in the first trimester, and one case was identified with fetal rhabdomyoma. In case 4,it was diagnosed after birth, primarily because at 24 weeks of gestation, the HC, FL, and HL were all below M-2SD. Due to the lack of prenatal indications for WES, the condition was not detected prenatally. After birth, WES performed on venous blood identified a pathogenic variant in the EP300 gene.

The spectrum of phenotypes associated with *EP300* variants, as documented in the literature, encompasses a wide range of abnormalities, including multiple malformations of the nervous system (such as posterior fossa hypoplasia and neural tube defects) (Hadzsiev et al., 2019), the urogenital system (such as kidney agenesis) (Solomon et al., 2015), and anorectal malformations (Masuda et al., 2015). In a study conducted by Yu et al. (Yu et al., 2025) in China, four fetuses with prenatally diagnosed *EP300* abnormalities exhibited phenotypes distinct from those observed in the current study. We compared our four EP300 cases with the four EP300 cases from Yu et al. Both cohorts commonly showed growth restriction (50% vs. 25%) and loss-of-function variants (three in each). Yu et al. reported congenital heart defects (right aortic arch, VSD) and polyhydramnios, whereas we found preliminary observations including rhabdomyoma, exencephaly, and limb postural abnormalities.

Clinical outcomes were similar (75% termination in both). The findings suggest that the spectrum of prenatal ultrasound phenotypes resulting from *EP300* haploinsufficiency is highly heterogeneous and extensive, ranging from severe, life-threatening structural malformations to isolated growth restriction. To systematically summarize the prenatal and postnatal phenotypes of RSTS2 cases with *de novo* pathogenic EP300 variants reported in the literature, we compiled studies meeting the inclusion criteria, as shown in Table 3. In summary, *EP300* haploinsufficiency leads to variable prenatal phenotypes. Our study identified new observed manifestations not previously reported by Yu et al. Additionally, WES may be considered for fetuses with unexplained growth restriction or any structural anomaly, even in the absence of classic thumb/toe deformities.

**TABLE 3:** Summary of prenatal and postnatal phenotypes in previously reported Rubinstein-Taybi syndrome cases carrying novel EP300 variants.

First author	Case	Variable sites	Prenatal ultrasound findings	Post-partum associated findings
Yu et al. (2025)	Case 9	c.277dup (p.Leu93Pfs*28)	VSD, CoA	TOP at 27 weeks
	Case 10	c.3863_3874+2del	FGR	TOP at 32 weeks
	Case 11	c.5610del (p.Q1870Hfs*36)	Polyhydramnios (AFI 29.7 cm)	Distinctive facial features Developmental delay
	Case 12	c.729 + 1_729+5del	RAA	TOP at 25 weeks
Zhang et al. (2025)	case 3	c.3764A>G (p.H1255R)	Polyhydramnios (AFI 28.9 cm) FGR	VSD, hypotonia Single transverse palmar creases
	Case 4	c.4452 + 5G>C	RAA, VSD, FGR	Low hairline, hypertrichosis Growth retardation
Negri et al. (2015)	Case 54	c.669dupT (p.Q223Sfs*19)	FGR	Prominent beaked nose, microcephaly, dowslanting palpebral Fissures, jaw, anomalies/micrognathia
	Case 133	c.3501 + 1G>A	FGR	Prominent beaked nose Microcephaly Columella below the alae nasi ‘Grimacing’ smile
Solomon et al. (2015)	​	c.4933C>T (p.Arg1645X)	Spinal anomaly	Microcephaly, hypoplastic left labia, absence of the left kidney
Masuda et al. (2015)	​	c. 2445delC (p.S815fs)	FGR	Anorectal malformations
Hadzsiev et al. (2019)	​	c.5698_5714delAAGGCAGCAGGCCAGGT (p.K1900Dfs*167)	NO	Hypoplasia of the inferior portion of the cerebellar vermis, tethered cord, mild syringohydromyelia
Fergelot et al. (2016)	Case 5	c.3857A > G (p.N1286S)	NO	Syringomelia
	Case 17	c.4933C > T (p.R1645*)	NO	Sacral lipoid myelomeningocele

VSD: ventricular septal defect, RAA: right aortic arch, CoA: coarctation of aorta, FGR: fetal growth restriction, AFI: amniotic fluid index, TOP: termination of pregnancy.

It is noteworthy that one fetus was prenatally diagnosed with rhabdomyoma, and WES revealed a variant in the *EP300*, with rhabdomyoma being the sole abnormal manifestation. Current literature on RSTS2 primarily associates cardiac abnormalities with aortic stenosis and atrioventricular septal defects (Reuter et al., 2021), with no prior reports linking rhabdomyoma to this condition. Our findings represent the first instance of fetal cardiac rhabdomyoma associated with a variant in the *EP300*, thereby providing additional evidence that *EP300* variants may predispose individuals to cardiovascular abnormalities. Additionally, in this study, a fetus diagnosed with exencephaly in the first trimester was confirmed via WES to carry a frameshift variant in the *EP300*. Previous literature on EP300-related central nervous system abnormalities has predominantly focused on cerebellar hypoplasia and dysplasia/agenesis of the corpus callosum, with severe open neural tube defects such as exencephaly being rarely reported. This case thus adds to the spectrum of neural tube developmental phenotypes potentially attributable to *EP300* haploinsufficiency during the prenatal period.

The literature on RSTS2 has reported a significantly higher incidence of preeclampsia in patients with this syndrome (Cohen et al., 2020; Fergelot et al., 2016). However, this study did not find such an incidence in pregnant women with high blood pressure. The primary reason may be the early diagnosis of gestational age in 1–3 cases and cesarean delivery due to fetal distress at 35 weeks of gestation in 4 cases, which may have precluded the observation of hypertensive conditions in the pregnant women. The four *EP300* variants identified in this study are novel and have not been previously recorded in the ClinVar and HGMD databases, thereby expanding the mutation spectrum of this gene. No specific prenatal ultrasound findings were observed. The most common clinical manifestation was the restriction of long bone growth during the second trimester, aligning with the prenatal diagnostic challenges highlighted by the International Consensus on the Diagnosis and Management of RSTS (Lacombe et al., 2024). In the absence of a family history or typical thumb/toe deformities, specific prenatal imaging signs are often lacking.

The limitations of this study include its limited sample size and the lack of functional studies on the novel variants identified on *EP300*. Future research should further investigate the pathogenic mechanisms of novel *EP300* variants, accumulate more case data, conduct multicenter studies, and establish a prenatal phenotypic spectrum of *EP300* variants for clinical application.

In conclusion, the reported prenatal ultrasound phenotypes associated with *EP300* variants may add to the phenotypic profile of RSTS2 and could serve as a reference for prenatal diagnosis and counseling. However, the diagnosis of RSTS2 based solely on ultrasound features remains challenging. We recommend detailed ultrasound evaluation in fetuses exhibiting growth restriction or structural anomalies. When conventional Karyotype and copy number variant analyses were negative, WES should be considered to aid in the detection of monogenic disorders, thereby supporting precise prenatal management and informed family counseling.

## Data Availability

The original contributions presented in the study are included in the article/supplementary material, further inquiries can be directed to the corresponding authors.
